# Circulating Nitrite in Severe Asthma: Just Another Biomarker or Novel Treatment Target?

**DOI:** 10.1111/all.16435

**Published:** 2024-12-19

**Authors:** Anna Freeman, Magdalena Minnion, Paul H. Lee, Hans Michael Haitchi, Ramesh Kurukulaaratchy, Tom Wilkinson, Martin Feelisch

**Affiliations:** ^1^ Clinical and Experimental Sciences, Faculty of Medicine University of Southampton Southampton UK; ^2^ National Institute for Health Research (NIHR) Southampton Biomedical Research Centre University Hospital Southampton NHS Foundation Trust Southampton UK; ^3^ Southampton Clinical Trials Unit University of Southampton Southampton UK; ^4^ The David Hide Asthma & Allergy Research Centre, St Mary's Hospital Newport UK

To the Editor,

Many patients with severe asthma do not meet biologic prescribing criteria in terms of exacerbation frequency, or do not respond fully to biologic therapies; consequently, significant symptom and exacerbation burdens remain. Novel treatment targets and interventions are needed for this group. Redox buffering modulates T2 inflammation, with nitrite a downstream marker [1, 2]. We here present novel data that implicate that circulating nitrite concentrations are associated with asthma inflammation and control.

Pilot data from our 12‐week structured exercise intervention demonstrated improved asthma symptoms and inflammation, alongside higher plasma nitrite levels following the intervention [1]. We demonstrated that increased physical fitness is strongly associated with elevations in steady‐state nitrite and antioxidant levels, with concomitant reductions in eosinophilic inflammation, and exercise‐induced upregulated circulating nitrite levels and improved redox buffering [1].

Nitrite has been studied most commonly in exhaled breath condensate (EBC) of severe asthmatics, although one study reported serum levels of nitrate to be higher in children with poorly controlled asthma [2]. Proof‐of‐principle administration of nebulised sodium nitrite in asthma patients demonstrated improvements in FEV1 and reduced exacerbations [3]. While oral nitrite upregulates antioxidant pathways [4] and improves mitochondrial fitness [5], there are no data demonstrating a link between nitrite levels in blood and asthma severity/symptom burden. In clinical practice, fractional exhaled nitric oxide (FeNO) is used as a marker of airways inflammation, yet the complex interaction between NO metabolism in a whole‐body system and asthma control remains unelucidated.

We hypothesised that severe asthma patients with high symptom and inflammation burden (poorly controlled) would demonstrate lower plasma nitrite than well‐controlled patients, alongside raised exacerbation frequency and asthma burden. To explore this, we identified 10 poorly controlled patients within the Wessex Asthma Cohort of Difficult Asthma (WATCH) [6] with peripheral blood eosinophils (PBEs), > 0.2, Asthma Control Questionnaire score (ACQ6) > 1.5 and ANCA negative (or not tested), plus 8 well‐controlled patients who met criteria of an eosinophil count ≤ 0.2 × 10 × 9/L and ACQ6 < 1 (see Supporting Information S1 for further details). Neither group were on maintenance oral corticosteroids or biologics. Inhaled corticosteroid (ICS) treatment was lower in the poorly controlled group; this is a reflection of the real‐life nature of the WATCH study, where data are captured alongside clinical care. Many of these patients are recruited at their first clinic visit, with appropriate up‐titration of ICS at that occasion. For this study, we only looked at baseline samples and data. Plasma was analysed for nitrite and nitrate as markers of NO formation/metabolism, the thiol metabolome (reduced/oxidised low‐molecular‐weight thiols such as glutathione, cysteine and sulfide), total free thiols (TFT), and antioxidant levels (ferric reducing antioxidant capacity of plasma; FRAP), as described previously [1], alongside clinical metadata. Lactate/pyruvate and 5‐oxoproline levels were assessed as markers of tissue NAD^+^/NADH and glycine availability, respectively. Data were not all normally distributed (Shapiro–Wilk test), and therefore nonparametric tests were used for analysis. Demographics are displayed in Table 1.

**TABLE 1:** Demographic data for WATCH participants.

Demographics	Poorly controlled (*n* = 10) *n* (%) or (median [IQR])	Well controlled (*n* = 8) *n* (%) or (median [IQR])
Age	52.5 (44.8, 66)	58 (44.5, 63.5)
Female	7 (70%)	7 (88%)
BMI (kg/m^2^)	27 (24.9, 33.7)	27.7 (20.5, 30)
Age of asthma onset^a^		
Childhood	5 (55%)	2 (29%)
Adult	4 (45%)	5 (71%)
Smoking history^a^		
Current	0	0
Former	5 (50%)	1 (14%)
Never	5 (50%)	6 (86%)
Clinical features		
Atopy^a^	5 (55%)	3 (60%)
FEV1 pre BD (%pred)^a^	85.5 (69.1, 102.6)	86.8 (97.6, 97.6)
FVC pre BD (%pred)^a^	107.8 (85.9, 117)	110.7 (115.8, 115.8)
FEV1/FVC post BD^a^	73 (47, 89.5)	63 (65, 65)
FeNO (ppb)	25.3 (11.7, 51)	14.9 (6.4, 51.8)
Blood eosinophil count (10^9^/L)	0.85 (0.7, 1.15)	0.05 (0.0, 0.175)
Asthma control		
ACQ 6	3 (1.8, 4.3)	0.75 (0.5, 0.8)
Exacerbations	5.5 (2.75, 6)	1 (0, 3)
Asthma treatment		
ICS dose (μg/day BDP equivalent)	1000 (1000, 1875)	2000 (2000,3000)

Abbreviations: ACQ6, Asthma Control Questionnaire; BD, bronchodilator; BDP, beclometasone dipropionate; BMI, body mass index; FeNO, fractional exhaled nitric oxide; FEV1, forced expiratory volume in 1 s; FVC, forced vital capacity; ICS, inhaled corticosteroids.

^a^ Missing data.

Plasma nitrite levels were significantly higher in patients with well‐controlled asthma (0.75 [0.61, 0.89] vs. 0.32 [0.18, 0.41], *p* = < 0.0001). While readouts of whole‐body redox balance (FRAP, TFT) were comparable between groups, ratios of reduced to oxidised glutathione and cysteine were lower in well‐controlled asthma (*p* = 0.03 and *p* = 0.01; Figure 1a–f). Higher plasma nitrite was associated with lower symptom burden (*r* = −0.694, *p* = 0.001), PBEs (*r* = −0.912, *p* < 0.001), total IgE (*r* = −0.569, *p* = 0.017), fewer exacerbations (*r* = −0.687, *p* = 0.002) and higher quality of life (*r* = −0.541, *p* = 0.037; Figure 1g–n). No significant correlations were observed between FeNO levels and ICS dose, nitrate, nitrite or FRAP (data not shown) nor were there significant differences in nitrate, sulphide and other small thiol, pyruvate/lactate and 5‐oxoproline levels (Figures S1–S4).

**FIGURE 1:**
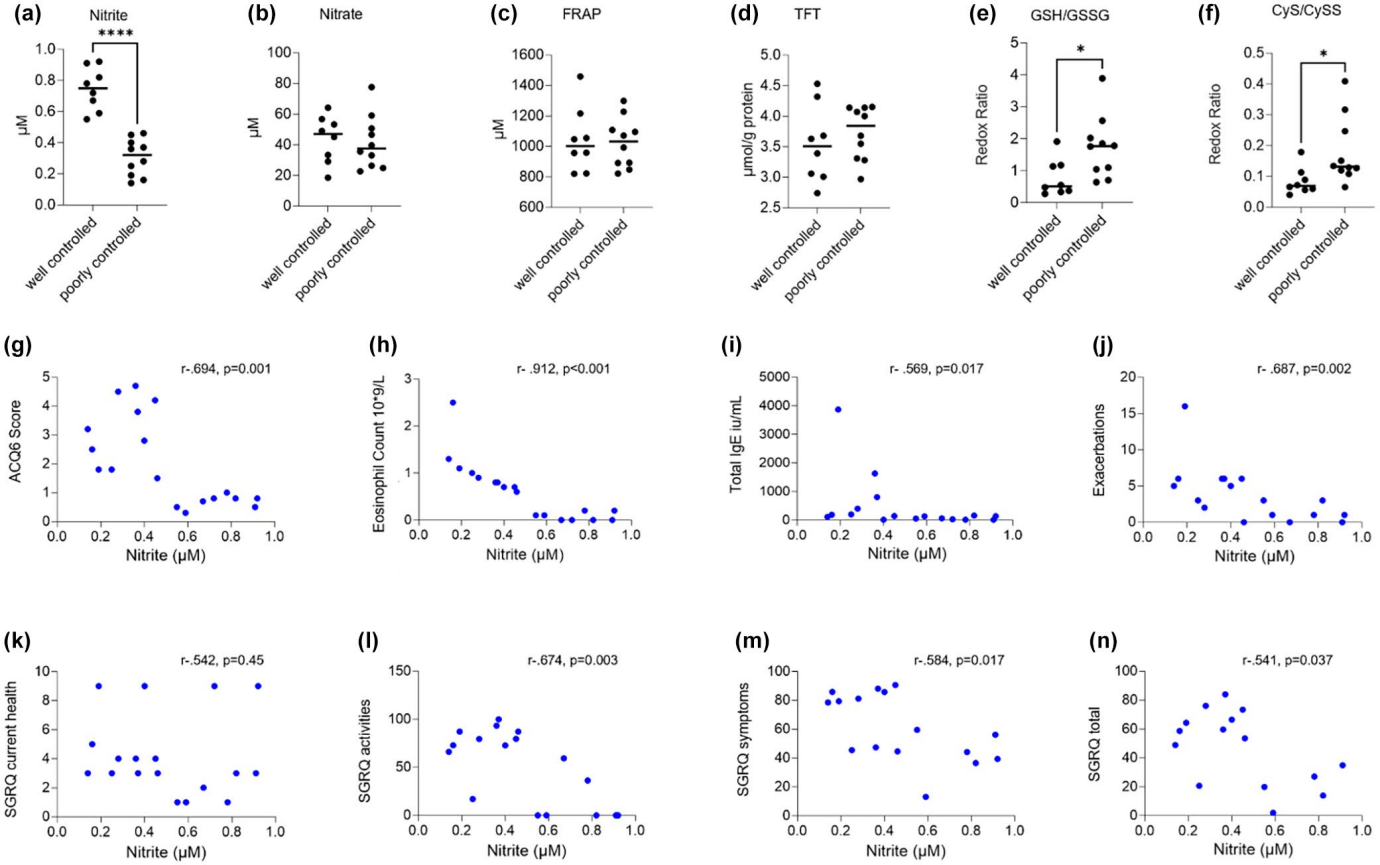
(a–j) Plasma nitrite, nitrate, antioxidant (ferric reducing ability of plasma; FRAP) and total free thiol (TFT) levels as well as redox ratios of glutathione and cysteine in patients with poorly and well‐controlled asthma, and significant correlations (Spearman's rho) between plasma nitrite levels and (g) ACQ6 score, (h) peripheral blood eosinophil count (10^9^/L), (j) Total IgE, (j) exacerbations requiring OCS in the past 12 months, (k) SGRQ current health, (l) SGRQ activities, (m) SGRQ symptoms and (n) total SGRQ score. ACQ, asthma control questionnaire; GSH, glutathione; GSSG, glutathione disulphide; OCS, oral corticosteroids; SGRQ, St George's Respiratory Questionnaire *p < 0.05, ****p < 0.0001.

We demonstrated previously that higher nitrite levels are associated with exercise‐related improvements in asthma control, redox buffering and eosinophilic inflammation [1], suggesting disease modulating potential. Our present study demonstrates that nitrite is a key marker of disease control in severe asthma, independent of whole‐body redox balance and local (FeNO) or systemic (nitrate) NO production. The lower redox ratios of glutathione and cysteine against the lack of differences in FRAP, TFT and other thiols highlight the complexity of redox interactions involved. Further investigations, either in in vivo animal or in vitro models, are warranted to determine mechanistic links between asthma symptoms, inflammation, physical activity, fitness and nitrite, with potential new opportunities for therapeutic modulation.

## Author Contributions

Authors A.F., M.M., H.M.H., R.K., T.W. and M.F. made substantial contributions to the conception and design of this work as well as data acquisition or analysis and interpretation and drafting the manuscript. P.H.L. contributed to the formal analysis of the final manuscript, providing statistical analysis and interpretation of the data. All authors were involved in revising the manuscript for important intellectual content, gave final approval of the version to be published and agreed to be accountable for all aspects of the work.

## Conflicts of Interest

The authors declare no conflicts of interest.

## Supporting information


Data S1.

Figure S1.
Figure S2.
Figure S3.
Figure S4.

## Data Availability

The data that support the findings of this study are available from the corresponding author upon reasonable request.
