# Supplementary material for: Circulating Nitrite in Severe Asthma: Just Another Biomarker or Novel Treatment Target?
Source: Allergy. 2024 Dec 19;80(4):1146–9. doi: 10.1111/all.16435 (PMC11969320; doi:10.1111/all.16435)
Supplement: Supplementary file 1 — Data S1. [file ALL-80-1146-s002.docx]

**Supplementary Information**

to

**Circulating Nitrite in Severe Asthma: Just Another Biomarker or Novel Treatment Target?**

Anna Freeman^1,2^, Magdalena Minnion^1,2^, Paul. H Lee^3^, Hans Michael Haitchi^1,2^, Ramesh Kurukulaaratchy^1,2,4^, Tom Wilkinson^1,2^, Martin Feelisch^1,2^

^1^ Clinical and Experimental Sciences, Faculty of Medicine, University of Southampton, UK.

^2^ National Institute for Health Research (NIHR) Southampton Biomedical Research Centre, University Hospital Southampton NHS Foundation Trust, Southampton, UK.

^3^ Southampton Clinical Trials Unit, University of Southampton, Southampton, UK

^4^ The David Hide Asthma & Allergy Research Centre, St Mary’s Hospital, Newport, Isle of Wight, UK.

*Corresponding author:*

Dr Anna Freeman

Mailpoint 810, F-Level, South Academic Block, Southampton General Hospital, Tremona Road, Southampton, SO16 6YD.

Telephone: 0238120 6397

Email: [a.freeman@soton.ac.uk](mailto:a.freeman@soton.ac.uk)

**Methods**

The Wessex Asthma Cohort of Difficult Asthma (WATCH) is a prospective observational study of severe asthma patients under the care of the University Hospital Southampton Severe Asthma Service, with specific methodology published in^(1)^. Aliquots of EDTA plasma samples, taken at enrolment, were selected from the WATCH Biobank (Approved by West Midlands – Solihull Research Ethics Committee (REC reference: 14/WM/1226), all patients gave written informed consent), with clinical metadata from the same timepoint^(1)^. Nitrate and nitrite concentrations were quantified using a dedicated high-performance liquid chromatography analysis system (ENO-30 with Insight autosampler, EiCom; Amuza Inc., San Diego, CA, USA), following sample deproteinization by methanol precipitation and centrifugation^(2)^. The concentrations of free and bound low-molecular-weight thiols including sulfide were quantified by ultrahigh pressure liquid chromatography-tandem mass spectrometry (UPLC-MS/MS), before and after reduction of plasma aliquots by dithiothreitol, as described^(3)^. Total free thiol (TFT) concentrations, which largely reflect the availability of the single free cysteine group (Cys-34) of serum albumin, were determined spectrophotometrically using Ellman’s reagent (5,5’-dithio-bis-2-nitrobenzoic acid; DTNB) and normalised to protein concentration, as described^(4)^. The ferric reducing ability of plasma (FRAP) was used as a measure of total antioxidant/reductive capacity^(5)^. This assay measures the reduction of ferric (Fe^3+^) to ferrous (Fe^2+^) ions by the formation of an intense, blue-coloured ferrous-tripyridyltriazine complex under acidic conditions; Fe^3+^ reacts with a variety of reducing compounds including uric and ascorbic acid, tocopherol and bilirubin, but not thiols. The combined readouts of TFT and FRAP were interpreted to reflect whole-body redox balance.

**Statistical Analysis**

This was an exploratory study and therefore not powered for significance. All data were treated as nonparametric, and data analysed on a per protocol basis. Significance was assumed if *p* < 0.05 using the Wilcoxon Signed-Rank Test or Friedman Test. Bivariate correlations between the redox metabolome and clinical markers of asthma were assessed using a Spearman’s test. An r value of >0.7 was considered a strong correlation, an r value of 0.4 to 0.7 was considered a moderate correlation. Repeated measures adjustments were not employed due to the small sample size and difficulty in accurately assessing data of this size for normal or non-normal distribution^(6)^. Calculations were performed using IBM SPSS 27 (IBM, Chicago, IL, USA) and GraphPad Prism 10 (GraphPad Software, San Diego, CA, USA), and figures were produced using GraphPad Prism.

**Supplementary Results and Discussion**

Both, nitrite (NO_2_^-^) and nitrate (NO_3_^-^) are products of the oxidative biotransformation of nitric oxide (NO) and are also contained in our diet, in particularly high concentration in green, leafy vegetables. Circulating nitrite concentrations are usually tightly controlled, reflecting constitutive vascular NO synthase activity^(7, 8)^. Independent of vascular endothelial function, it appears nitrite is directly linked with asthma control, and this is relatively independent of other markers of redox metabolism. The significant differences in the redox ratio of glutathione and cysteine between well controlled and poorly controlled groups is also noteworthy (Figure 1). The lower ratio in the well-controlled groups was unexpected and reflects a greater proportion of oxidised (GSSG and CySS) to reduced (GSH and Cys) thiols (Figure 1), which would suggest a higher level of oxidative stress, greater leakage of disulfides from cells/tissue, and/or an impaired reductive poise. GSH/GSSG ratio has been explored in the airways, using epithelial lining fluid, in children with severe asthma. Here, the redox potential, calculated using the Nernst equation, was lower in adult and child controls (i.e. more reduced GSH and less oxidised GSSG), with the greatest oxidation seen in those with severe asthma and airways obstruction^(9)^. The oxidation product of cysteine, cystine (Cyss) has been quantified systemically in children with asthma and compared to healthy controls, with higher levels of oxidation seen in asthma patients. Higher levels of cystine were associated with higher inhaled corticosteroid (ICS) dose, higher short-acting bronchodilator use, higher ACQ score, and more frequent emergency department attendances and intubations^(10)^. Furthermore, cysteine oxidation impaired glucocorticoid responsiveness to triamcinolone treatment in these patients^(10)^. Plasma GSSG and reactive oxygen species (ROS) generation was also higher in asthma patients compared to controls^(10)^. The differences in cysteine redox ratio seen between well controlled and poorly controlled in our cohort could be explained by differences in ICS, with those who were well controlled taking a higher dose of ICS and a significant negative correlation seen between ICS dose and Cys/Cyss ratio (r=-.888, p<0.001, data not presented), although this is reflective of better control in our cohort. Nitrite levels demonstrated a negative association with Cys/Cyss ratio (r=-.474, p=0.047, data not presented). Smoking status and atopy did not have any significant association with nitrate, nitrite and FRAP levels, with only age at onset demonstrating association with nitrate levels (data not shown). As with any study based in an observational cohort, there are some limitations to this pilot data. There are occasional missing data points due to the real-life, clinic integrated model of WATCH. The ICS dose, as above, for well controlled patients was higher than those who were poorly controlled; this may have an impact on redox markers. However, it does not detract from the clinical and/or mechanistic significance of our observation, which may, in the future, enable us to identify new treatment markers that can be targeted to allow lower dosing of ICS. The SHAMAL study demonstrated that whilst biologics allow reduction in ICS dose, removal of ICS treatment even in the context of good control in biologic treated patients can result in insidious lung function decline without impact on symptoms or exacerbation frequency ^(11)^. Identification of new targets that are steroid responsive may allow further reduction in ICS dose with development of new redox targeted treatments. Irrespective of the limitations of this work or the complexity of redox regulation, what is apparent is that the role of nitrite in asthma in these interactions appears central and warrants further investigation.

**Supplementary Figure Legends**

Supplementary Figure 1: Non-significant results for thiol ratios. Abbreviations HCyS; homocysteine, HCySS; homocystine, GSH; glutathione, CySS; cystine.

Supplementary Figure 2: Non-significant differences in levels of free thiols in well and poorly controlled asthma. Abbreviations CyS; Cysteine, Cys-Gly; cysteinyl-glycine, CySS; cystine, Glu-CyS; ɣ-glutamyl-L-cysteine, GSH; glutathione, GSSG; glutathione disulphide, HCyS; homocysteine, HCySS; homocystine, NAC; N-acetylcysteine, μM; micromolar.

Supplementary Figure 3: Non-significant differences between levels of total thiols between well and poorly controlled asthma. Abbreviations CyS; Cysteine, Cys-Gly; cysteinyl-glycine, Glu-CyS; Glutamyl-L-cysteine, GSH; glutathione, NAC; N-acetylcysteine, μM; micromolar.

Supplementary Figure 4: Non-significant differences between levels of pyruvate, lactate and pyroglutamate (5-oxoproline) between well and poorly controlled asthma. Abbreviations: μM; micromolar.

**Supplementary References**

1. Azim A, Mistry H, Freeman A, Barber C, Newell C, Gove K, et al. Protocol for the Wessex AsThma CoHort of difficult asthma (WATCH): a pragmatic real-life longitudinal study of difficult asthma in the clinic. BMC Pulm Med. 2019;19(1):99.

2. Mencke R, Al Ali L, de Koning MLY, Pasch A, Minnion M, Feelisch M, et al. Serum Calcification Propensity Is Increased in Myocardial Infarction and Hints at a Pathophysiological Role Independent of Classical Cardiovascular Risk Factors. Arterioscler Thromb Vasc Biol. 2024.

3. Sutton TR, Minnion M, Barbarino F, Koster G, Fernandez BO, Cumpstey AF, et al. A robust and versatile mass spectrometry platform for comprehensive assessment of the thiol redox metabolome. Redox Biol. 2018;16:359-80.

4. Koning AM, Meijers WC, Pasch A, Leuvenink HGD, Frenay AS, Dekker MM, et al. Serum free thiols in chronic heart failure. Pharmacol Res. 2016;111:452-8.

5. Benzie IF, Strain JJ. The ferric reducing ability of plasma (FRAP) as a measure of "antioxidant power": the FRAP assay. Anal Biochem. 1996;239(1):70-6.

6. Oberfeld D, Franke T. Evaluating the robustness of repeated measures analyses: the case of small sample sizes and nonnormal data. Behav Res Methods. 2013;45(3):792-812.

7. Kleinbongard P, Dejam A, Lauer T, Rassaf T, Schindler A, Picker O, et al. Plasma nitrite reflects constitutive nitric oxide synthase activity in mammals. Free Radic Biol Med. 2003;35(7):790-6.

8. Lauer T, Preik M, Rassaf T, Strauer BE, Deussen A, Feelisch M, et al. Plasma nitrite rather than nitrate reflects regional endothelial nitric oxide synthase activity but lacks intrinsic vasodilator action. Proc Natl Acad Sci U S A. 2001;98(22):12814-9.

9. Fitzpatrick AM, Teague WG, Holguin F, Yeh M, Brown LA. Airway glutathione homeostasis is altered in children with severe asthma: evidence for oxidant stress. J Allergy Clin Immunol. 2009;123(1):146-52.e8.

10. Stephenson ST, Brown LA, Helms MN, Qu H, Brown SD, Brown MR, et al. Cysteine oxidation impairs systemic glucocorticoid responsiveness in children with difficult-to-treat asthma. J Allergy Clin Immunol. 2015;136(2):454-61.e9.

11. Jackson DJ, Heaney LG, Humbert M, Kent BD, Shavit A, Hiljemark L, et al. Reduction of daily maintenance inhaled corticosteroids in patients with severe eosinophilic asthma treated with benralizumab (SHAMAL): a randomised, multicentre, open-label, phase 4 study. Lancet. 2024;403(10423):271-81.
