# Supplementary material for: Circulating Nitrite in Severe Asthma: Just Another Biomarker or Novel Treatment Target?
Source: Allergy. 2024 Dec 19;80(4):1146–9. doi: 10.1111/all.16435 (PMC11969320; doi:10.1111/all.16435)
Supplement: Supplementary file 2 — Figure S1. Figure S2. Figure S3. Figure S4. [file ALL-80-1146-s001.pdf]

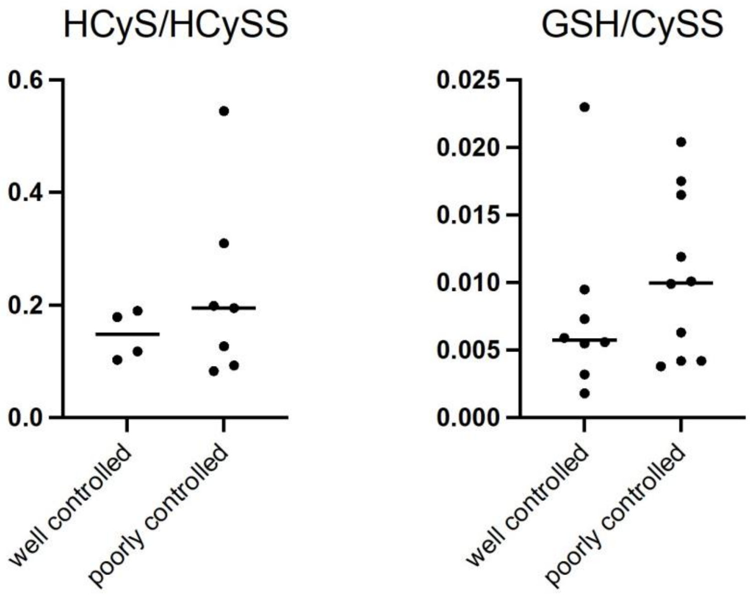

Supplementary Figure 1_Freeman et al.

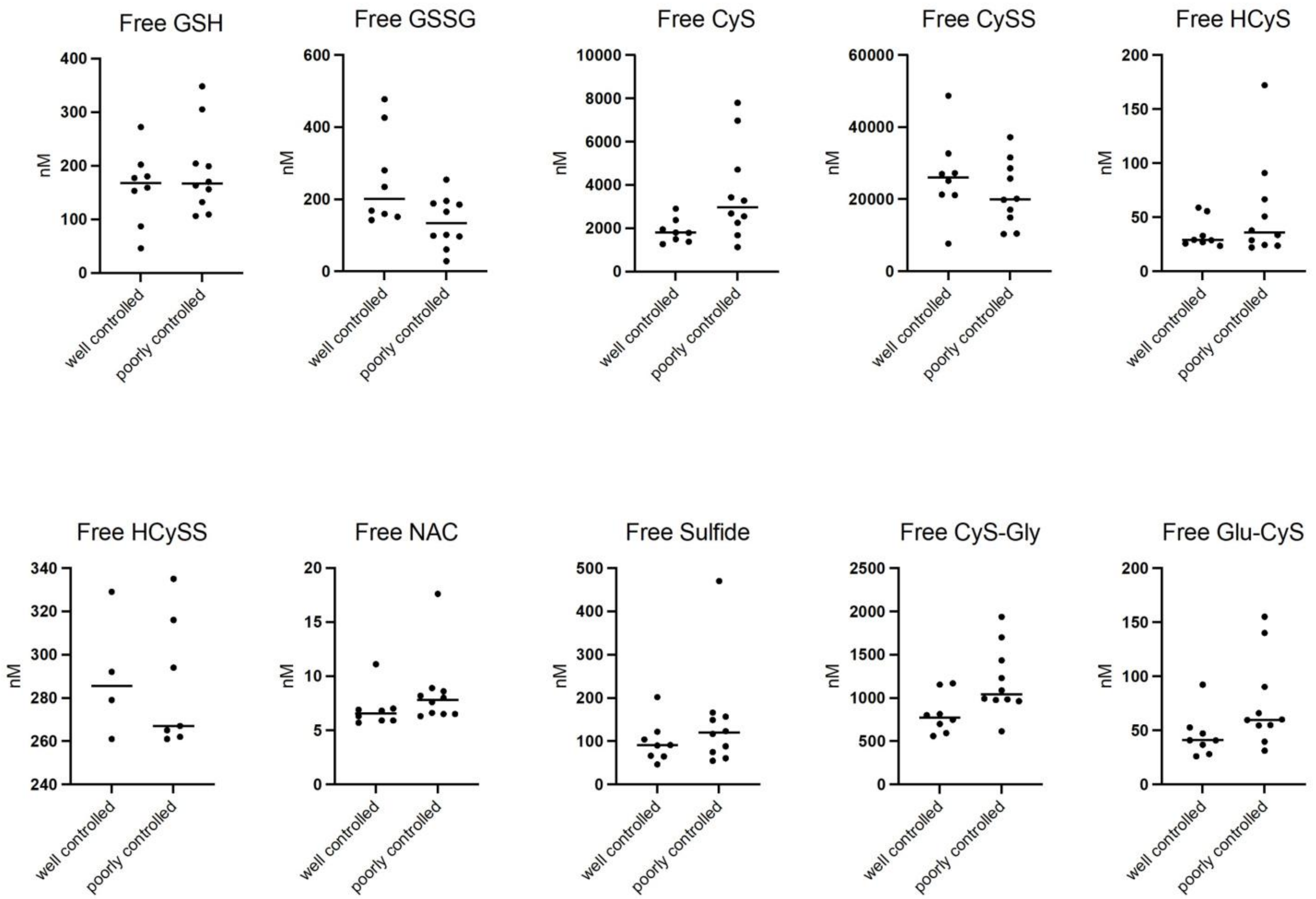

Supplementary Figure 2_Freeman et al.

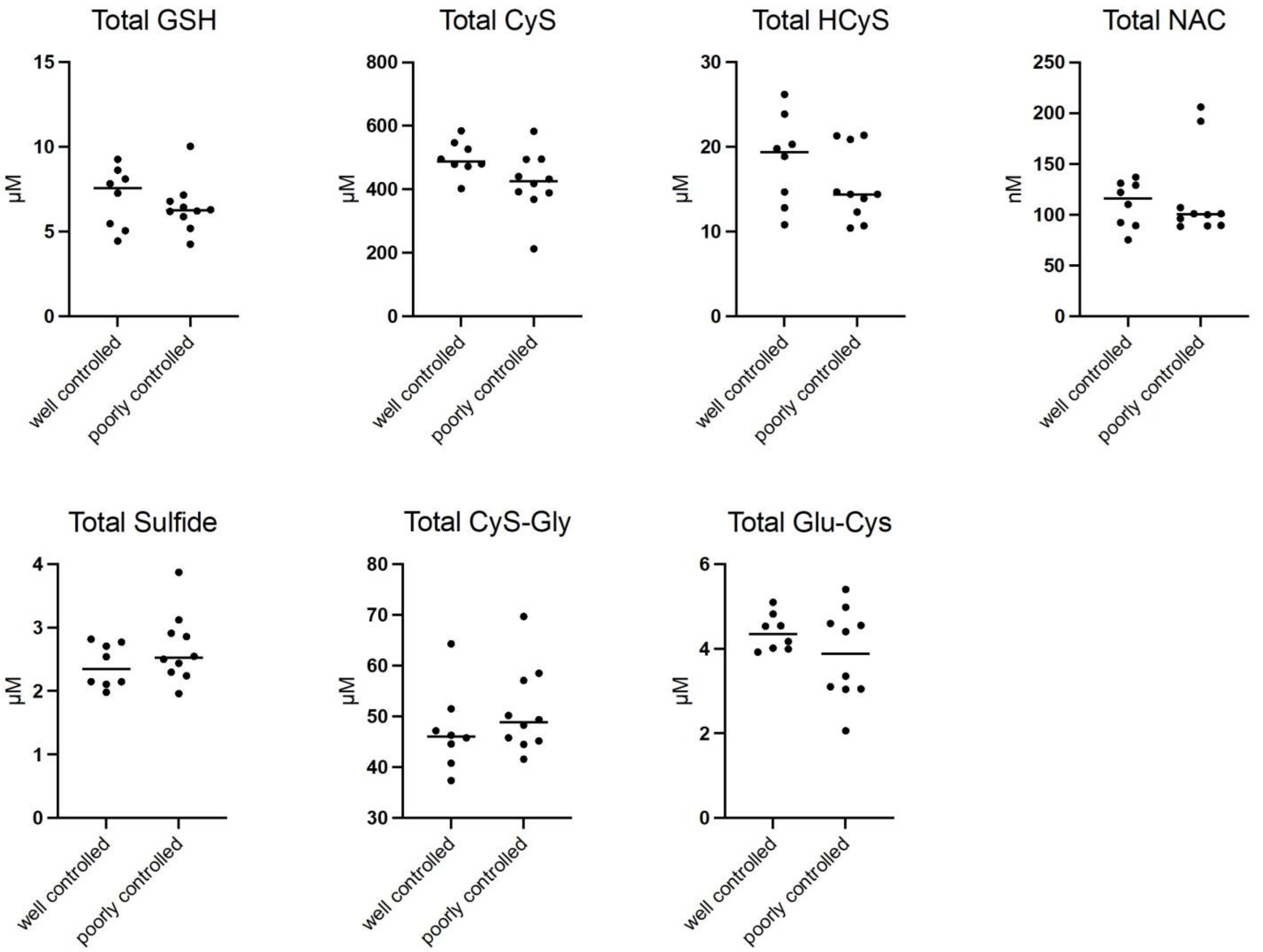

Supplementary Figure 3_Freeman et al.

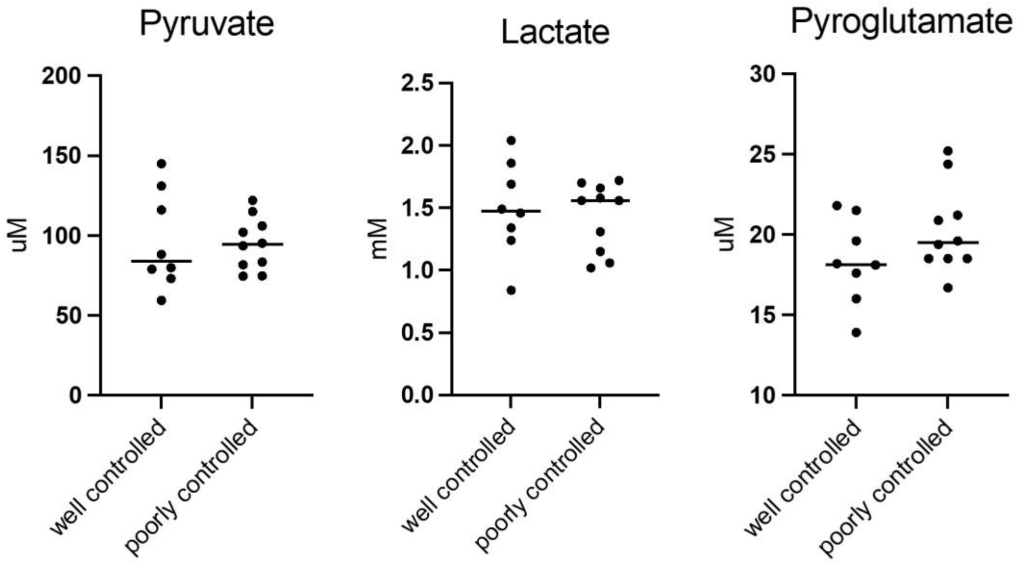

Supplementary Figure 4_Freeman et al.
